# Supplementary material for: Extracellular vesicles are the main contributor to the non-viral protected extracellular sequence space
Source: ISME Commun. 2023 Oct 17;3:112. doi: 10.1038/s43705-023-00317-6 (PMC10582014; doi:10.1038/s43705-023-00317-6)
Supplement: Supplementary file 2 — Figure S5 [file 43705_2023_317_MOESM2_ESM.pdf]

Figure S5 - Coverage Plots

Binned (bin size = 1000 bp) coverage plots of all analyzed MAGs.

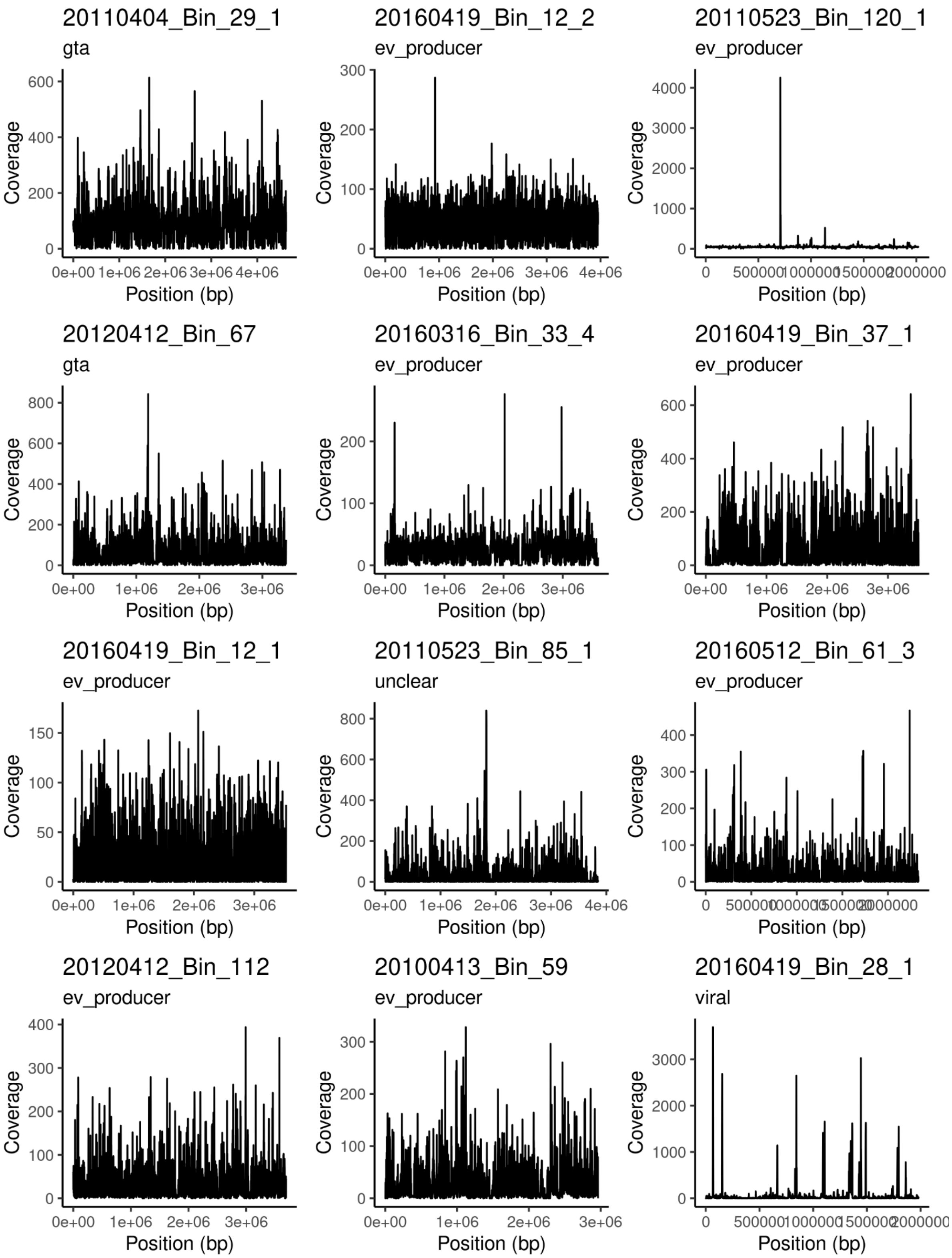
20110404_Bin_29_1
gta
20160419_Bin_12_2
ev_producer
20110523_Bin_120_1
ev_producer
20120412_Bin_67
gta
20160316_Bin_33_4
ev_producer
20160419_Bin_37_1
ev_producer
20160419_Bin_12_1
ev_producer
20110523_Bin_85_1
unclear
20160512_Bin_61_3
ev_producer
20120412_Bin_112
ev_producer
20100413_Bin_59
ev_producer
20160419_Bin_28_1
viral
Coverage
Position (bp)
0e+00
1e+06
2e+06
3e+06
4e+06
0
500000
1000000
1500000
2000000
0
50
100
150
200
300
400
600
800
1000
2000
3000
4000

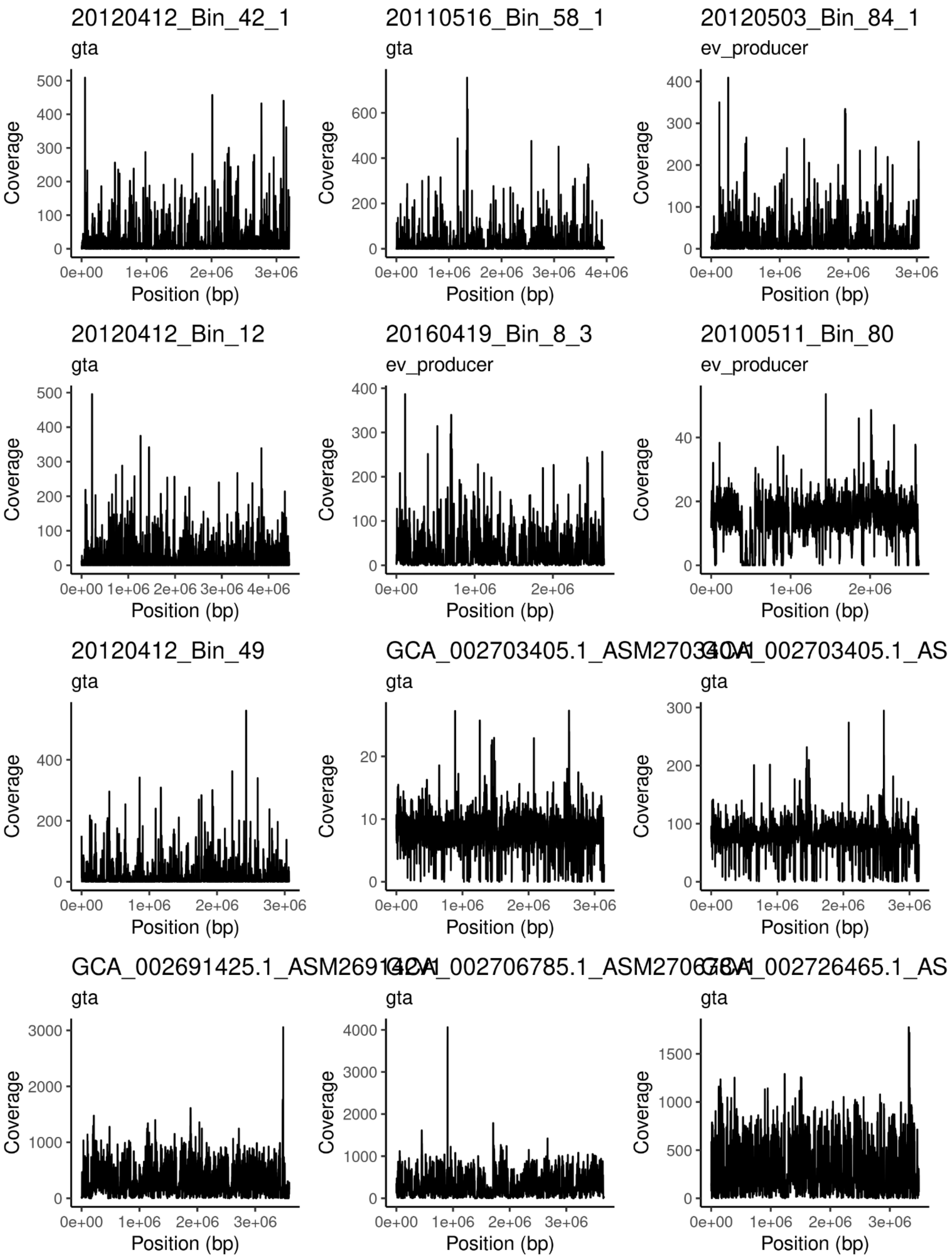
20120412_Bin_42_1
gta
20110516_Bin_58_1
gta
20120503_Bin_84_1
ev_producer
20120412_Bin_12
gta
20160419_Bin_8_3
ev_producer
20100511_Bin_80
ev_producer
20120412_Bin_49
gta
GCA_002703405.1_ASM270340v1
gta
GCA_002703405.1_AS
gta
GCA_002691425.1_ASM269142v1
gta
GCA_002706785.1_ASM270678v1
gta
GCA_002726465.1_AS
gta
Coverage
Position (bp)

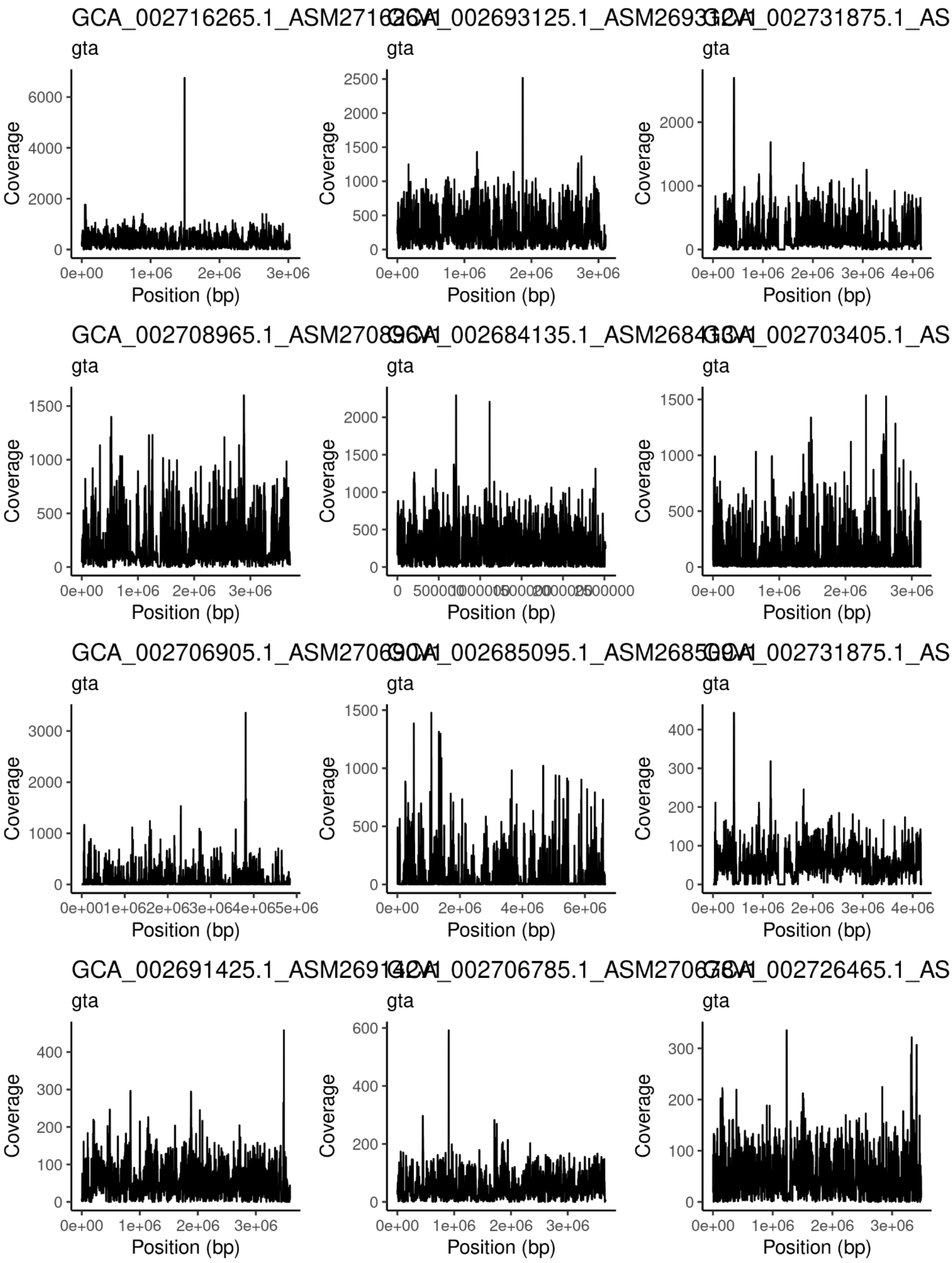

GCA_002716265.1_ASM2716
gta
Coverage
Position (bp)
002693125.1_ASM2693
gta
002731875.1_AS
gta
GCA_002708965.1_ASM2708
002684135.1_ASM2684
002703405.1_AS
GCA_002706905.1_ASM2706
002685095.1_ASM2685
002731875.1_AS
GCA_002691425.1_ASM2691
002706785.1_ASM2706
002726465.1_AS

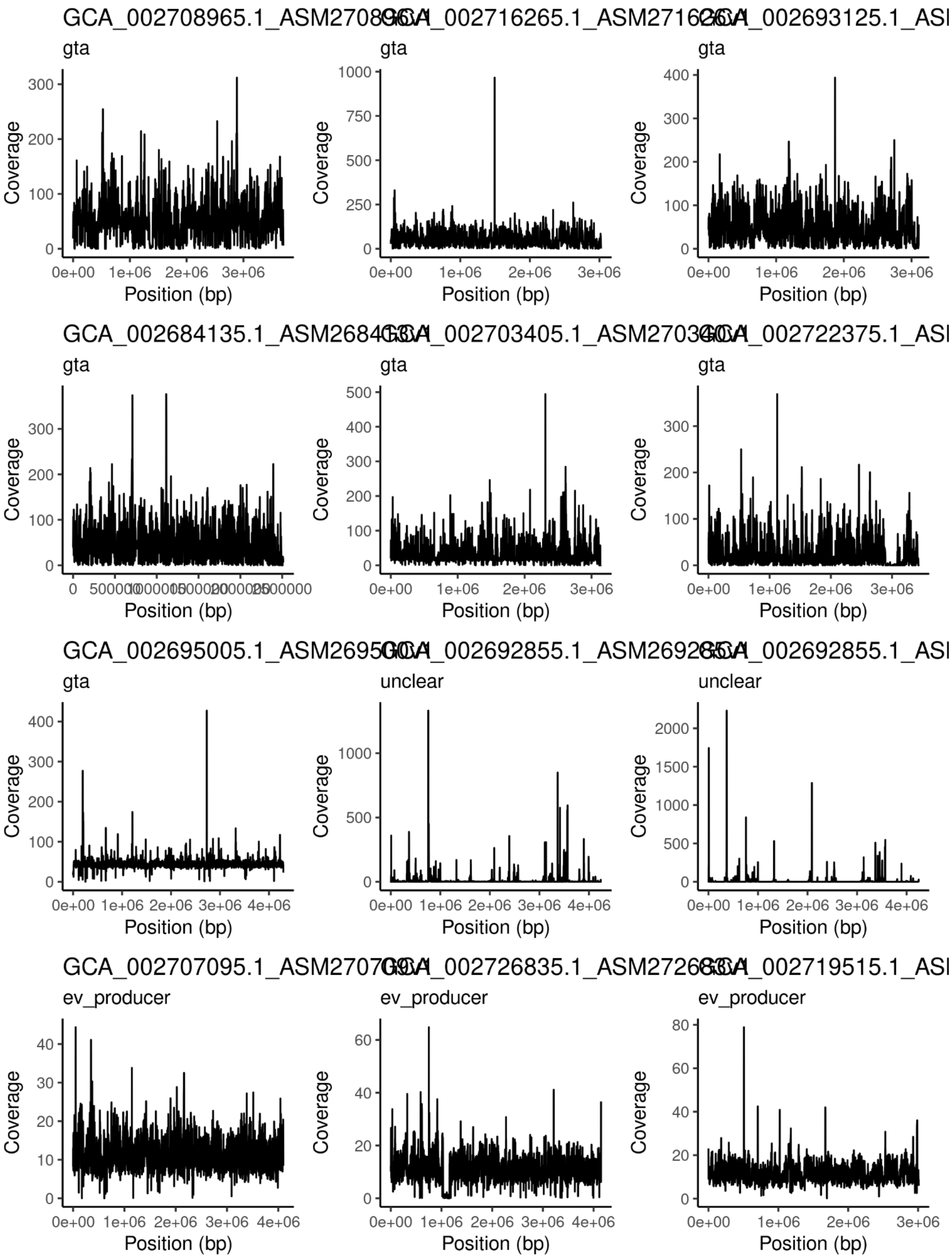
GCA_002708965.1_ASM27089
GCA_002716265.1_ASM2716
GCA_002693125.1_ASI
gta
GCA_002684135.1_ASM2684
GCA_002703405.1_ASM2703
GCA_002722375.1_ASI
gta
GCA_002695005.1_ASM2695
gta
GCA_002692855.1_ASM2692
GCA_002692855.1_ASI
unclear
GCA_002707095.1_ASM2707
GCA_002726835.1_ASM2726
GCA_002719515.1_ASI
ev_producer
Coverage
Position (bp)

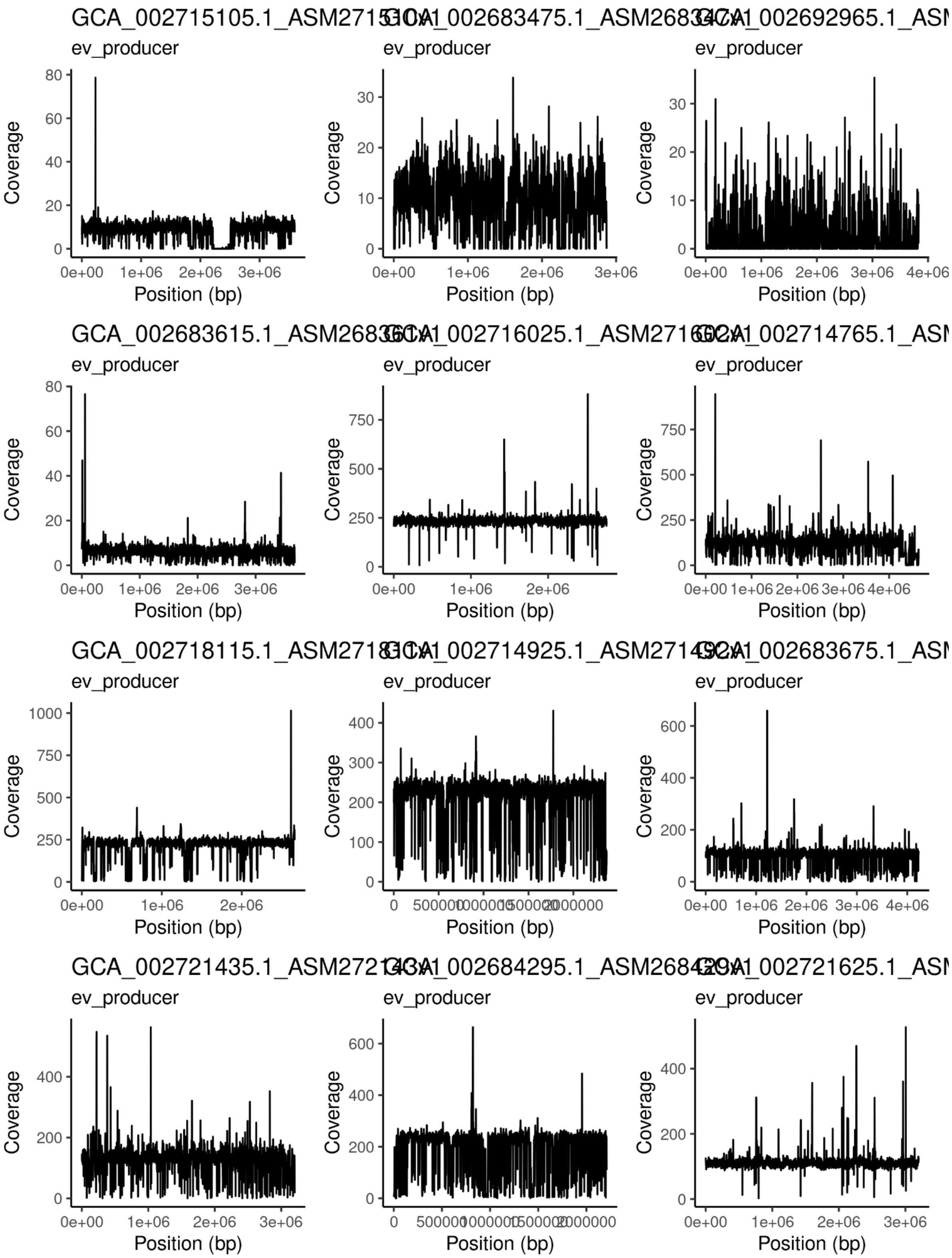

GCA_002715105.1
GCA_002683475.1
GCA_002692965.1
GCA_002683615.1
GCA_002716025.1
GCA_002714765.1
GCA_002718115.1
GCA_002714925.1
GCA_002683675.1
GCA_002721435.1
GCA_002684295.1
GCA_002721625.1
ev_producer
Coverage
Position (bp)

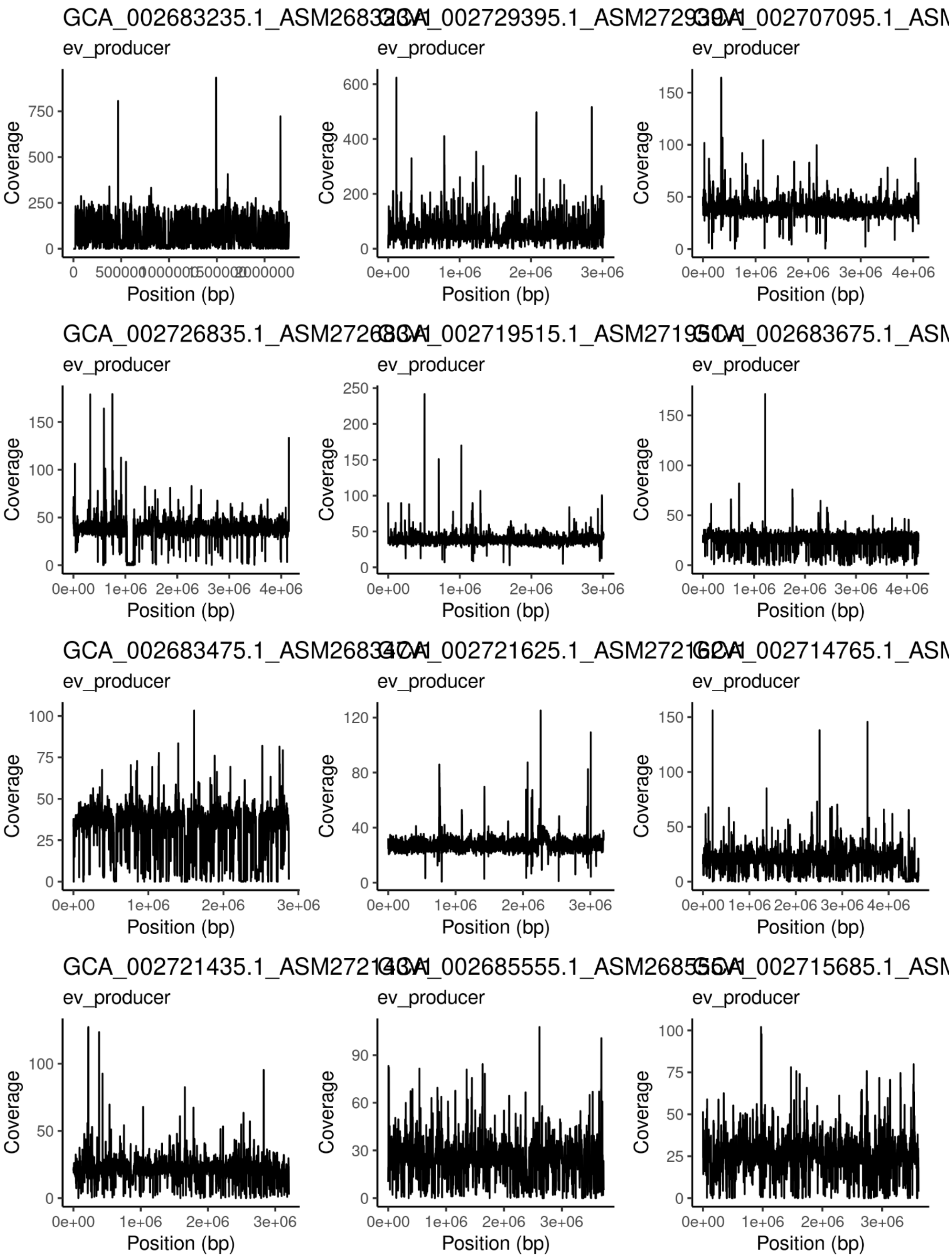
GCA_002683235.1_ASM268
GCA_002729395.1_ASM272
GCA_002707095.1_ASM
GCA_002726835.1_ASM272
GCA_002719515.1_ASM271
GCA_002683675.1_ASM
GCA_002683475.1_ASM268
GCA_002721625.1_ASM272
GCA_002714765.1_ASM
GCA_002721435.1_ASM272
GCA_002685555.1_ASM268
GCA_002715685.1_ASM
ev_producer
Coverage
Position (bp)

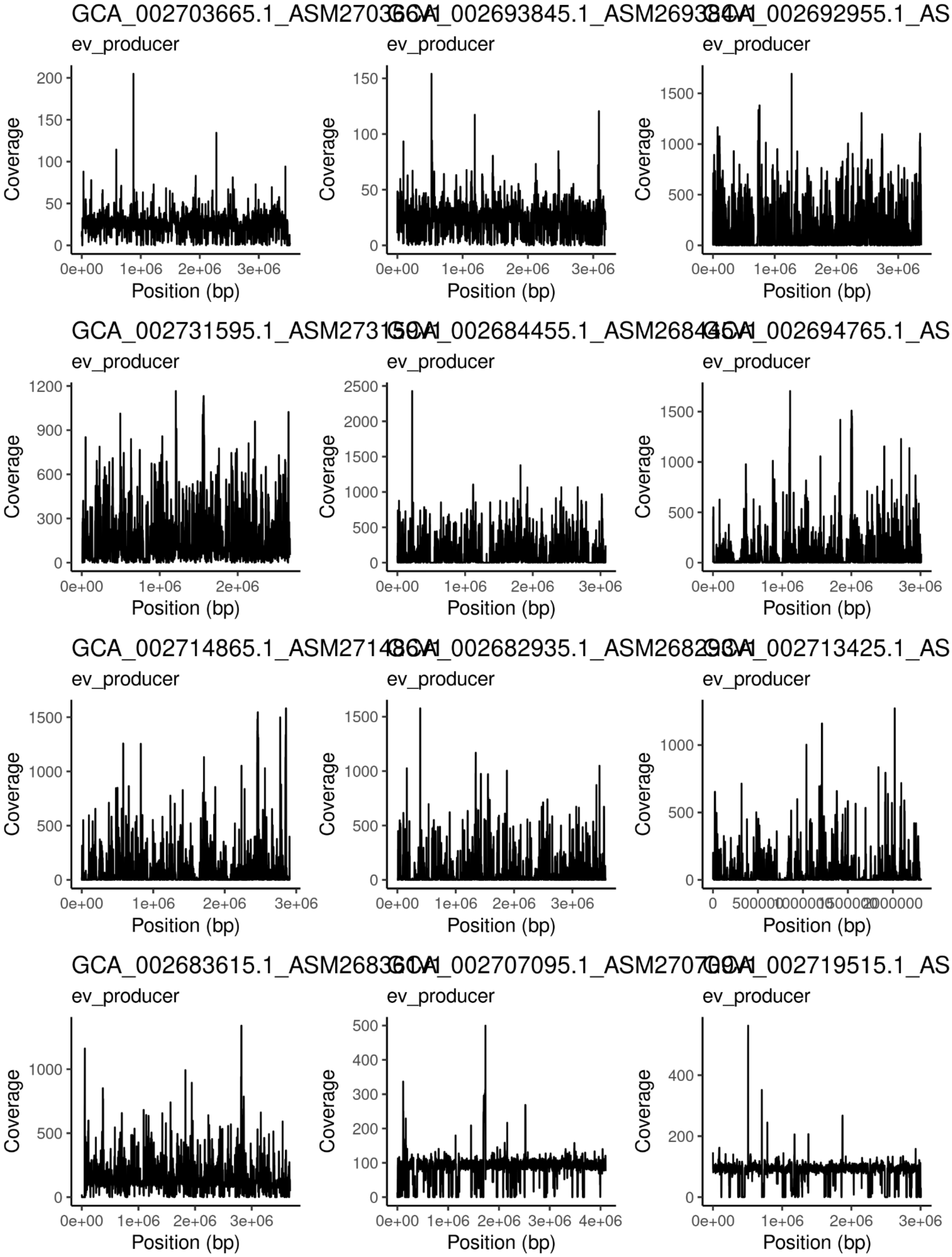

GCA_002703665.1_ASM2703
GCA_002693845.1_ASM2693
GCA_002692955.1_AS
GCA_002731595.1_ASM2731
GCA_002684455.1_ASM2684
GCA_002694765.1_AS
GCA_002714865.1_ASM2714
GCA_002682935.1_ASM2682
GCA_002713425.1_AS
GCA_002683615.1_ASM2683
GCA_002707095.1_ASM2707
GCA_002719515.1_AS
ev_producer
Coverage
Position (bp)

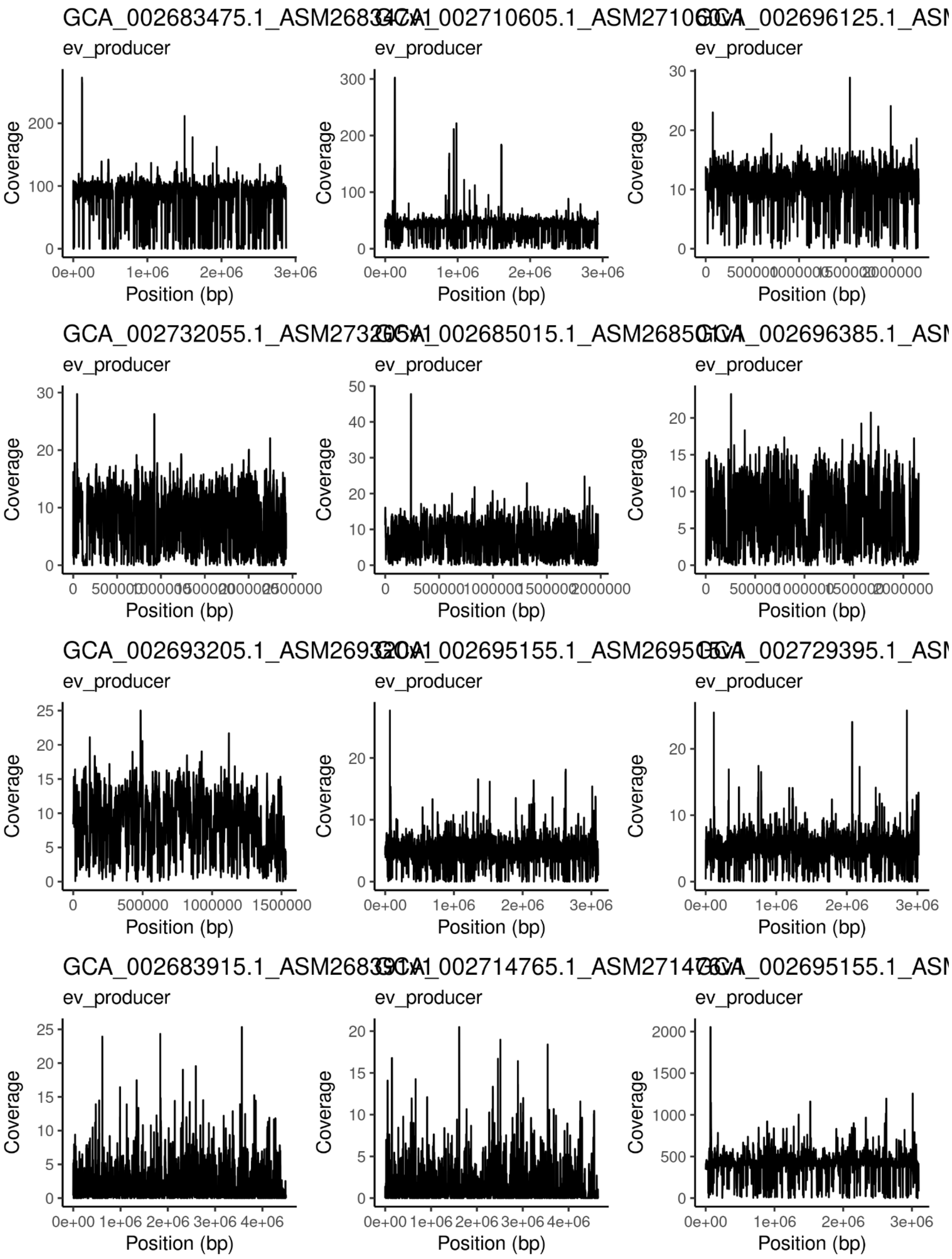

GCA_002710605.1_ASM27106
GCA_002696125.1_ASM
GCA_002685015.1_ASM2685
GCA_002696385.1_ASM
GCA_002693205.1_ASM2693
GCA_002695155.1_ASM2695
GCA_002729395.1_ASM
GCA_002714765.1_ASM27147
GCA_002695155.1_ASM
ev_producer
Coverage
Position (bp)

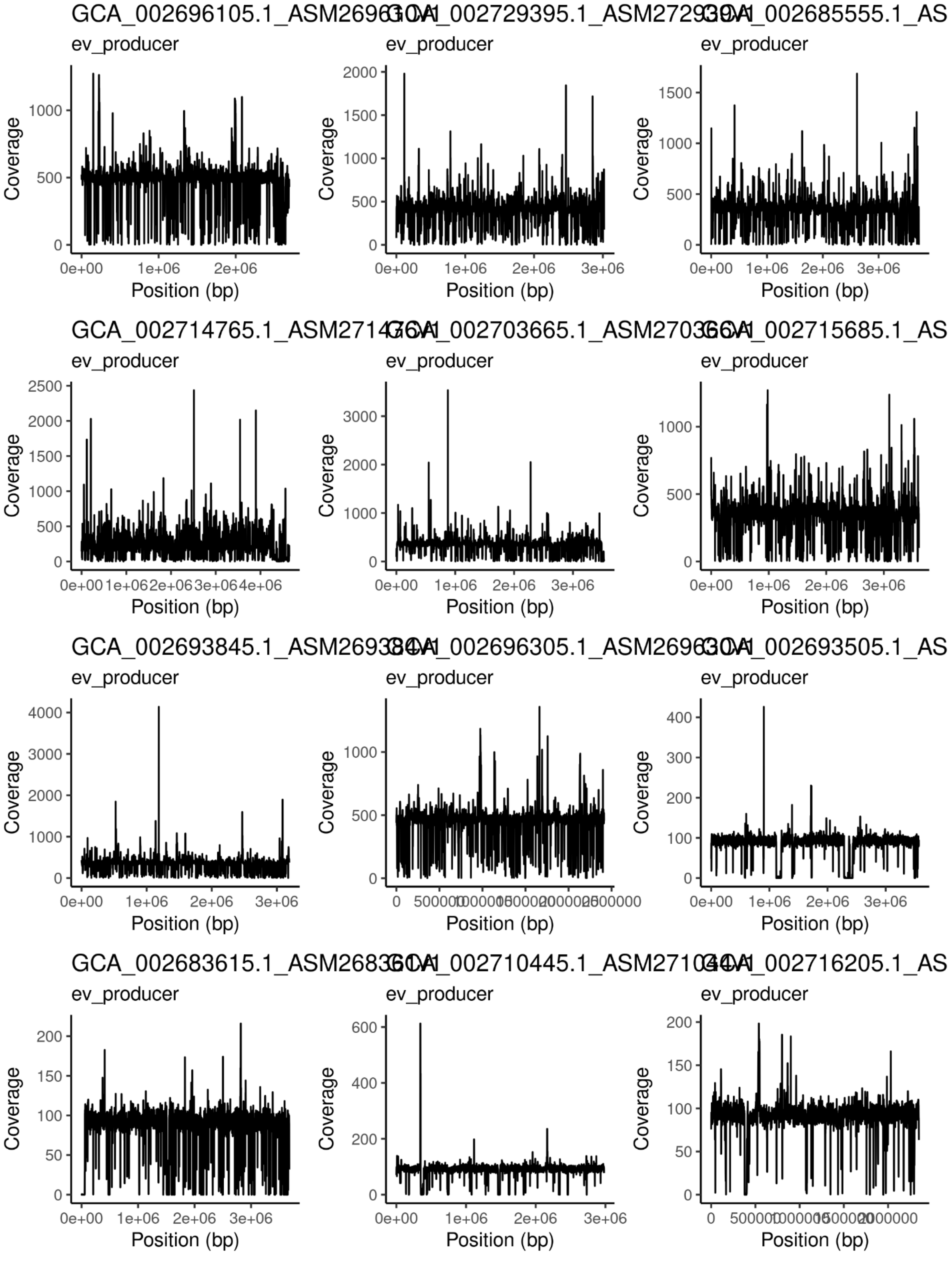

GCA_002696105.1
ev_producer
GCA_002685555.1_AS
GCA_002714765.1
GCA_002715685.1_AS
GCA_002693845.1
GCA_002693505.1_AS
GCA_002683615.1
GCA_002716205.1_AS
Coverage
Position (bp)

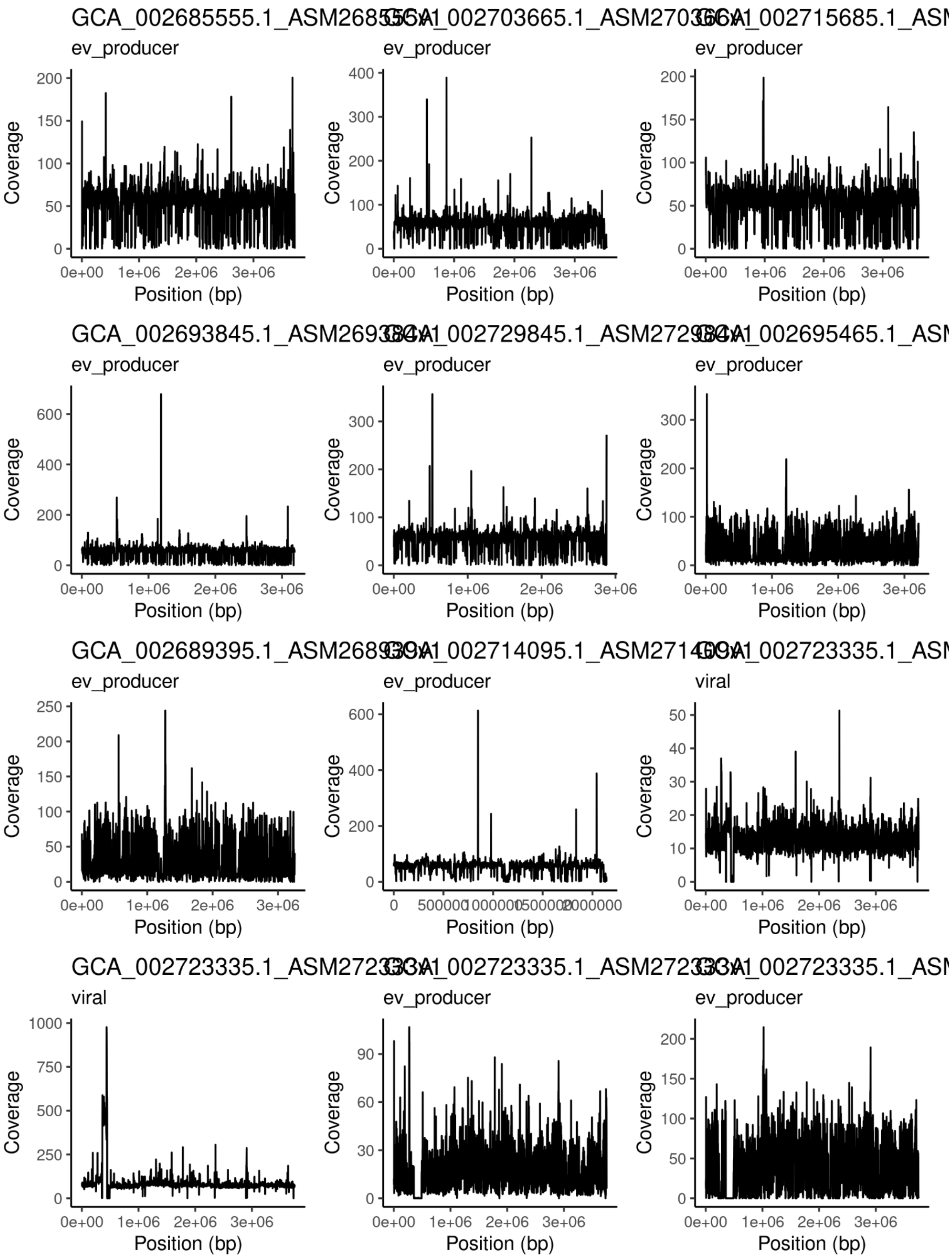

GCA_002685555.1
ev_producer
GCA_002703665.1
ev_producer
GCA_002715685.1
ev_producer
GCA_002693845.1
ev_producer
GCA_002729845.1
ev_producer
GCA_002695465.1
ev_producer
GCA_002689395.1
ev_producer
GCA_002714095.1
ev_producer
GCA_002723335.1
viral
GCA_002723335.1
viral
GCA_002723335.1
ev_producer
GCA_002723335.1
ev_producer
Coverage
Position (bp)

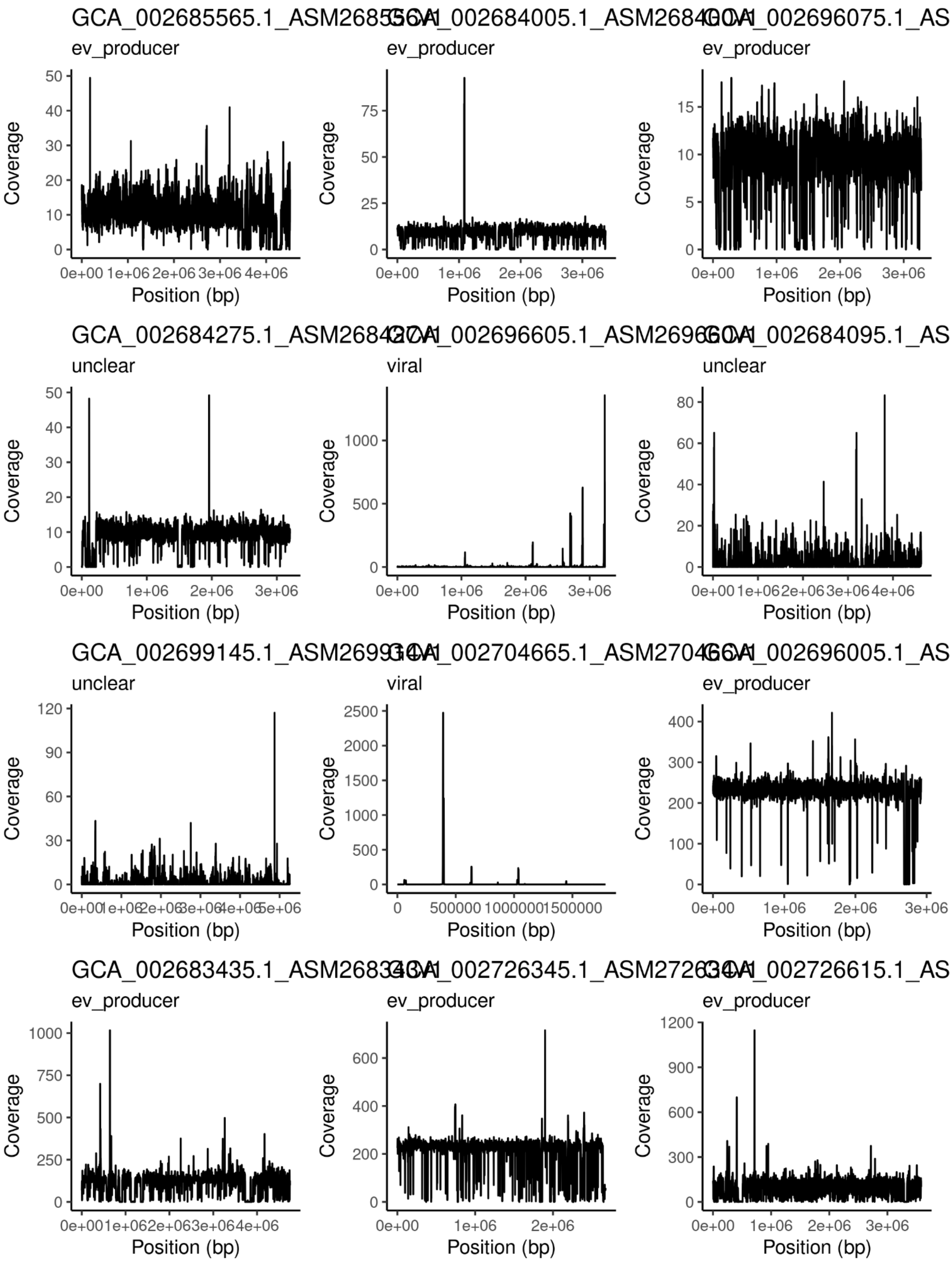

GCA_002685565.1_ASM2685
ev_producer
GCA_002684005.1_ASM2684
ev_producer
GCA_002696075.1_AS
ev_producer
GCA_002684275.1_ASM2684
unclear
GCA_002696605.1_ASM2696
viral
GCA_002684095.1_AS
unclear
GCA_002699145.1_ASM2699
unclear
GCA_002704665.1_ASM2704
viral
GCA_002696005.1_AS
ev_producer
GCA_002683435.1_ASM2683
ev_producer
GCA_002726345.1_ASM2726
ev_producer
GCA_002726615.1_AS
ev_producer
Coverage
Position (bp)

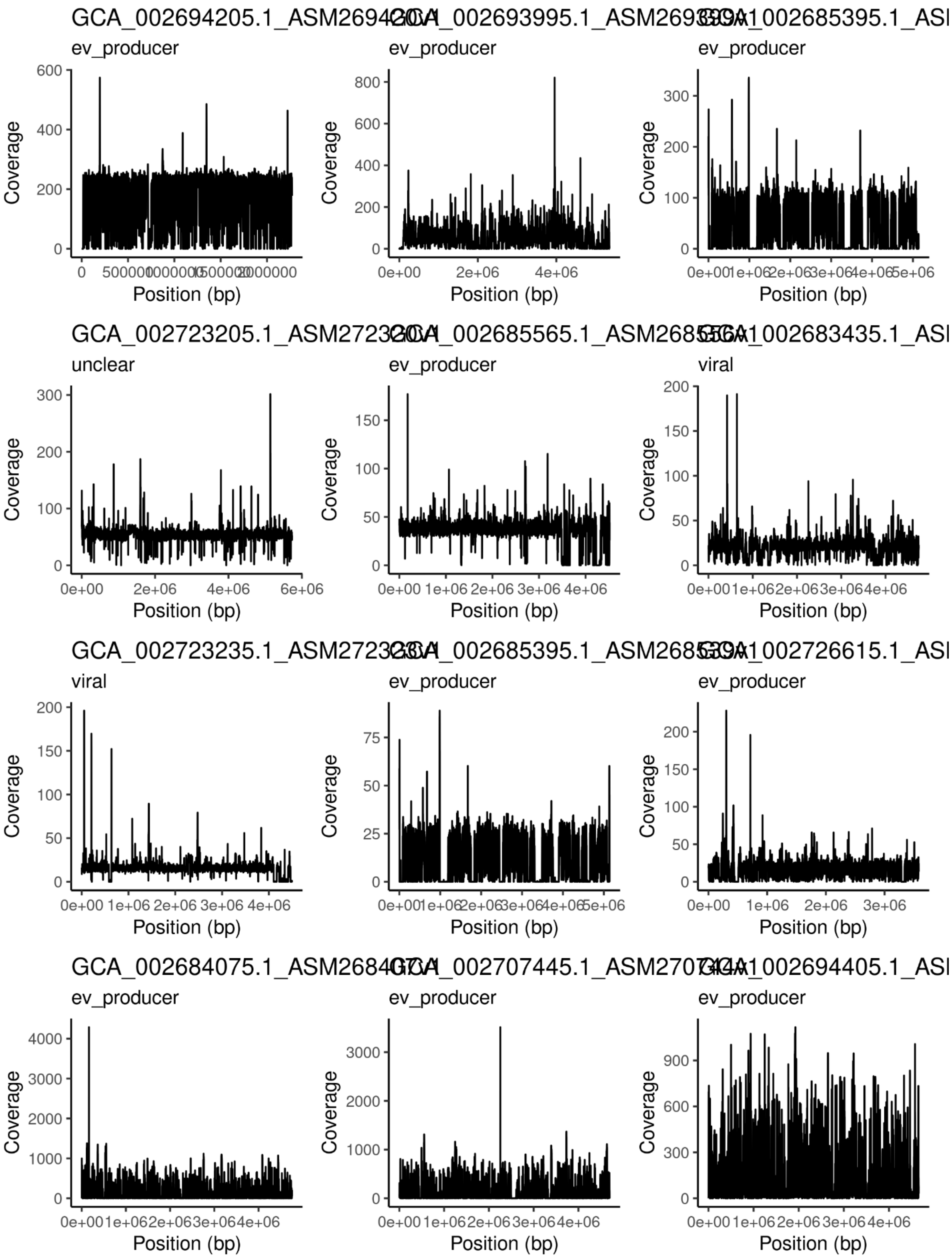

GCA_002694205.1
ev_producer
GCA_002693995.1
ev_producer
002685395.1_ASI
ev_producer
GCA_002723205.1
unclear
GCA_002685565.1
ev_producer
002683435.1_ASI
viral
GCA_002723235.1
viral
GCA_002685395.1
ev_producer
002726615.1_ASI
ev_producer
GCA_002684075.1
ev_producer
GCA_002707445.1
ev_producer
002694405.1_ASI
ev_producer
Coverage
Position (bp)

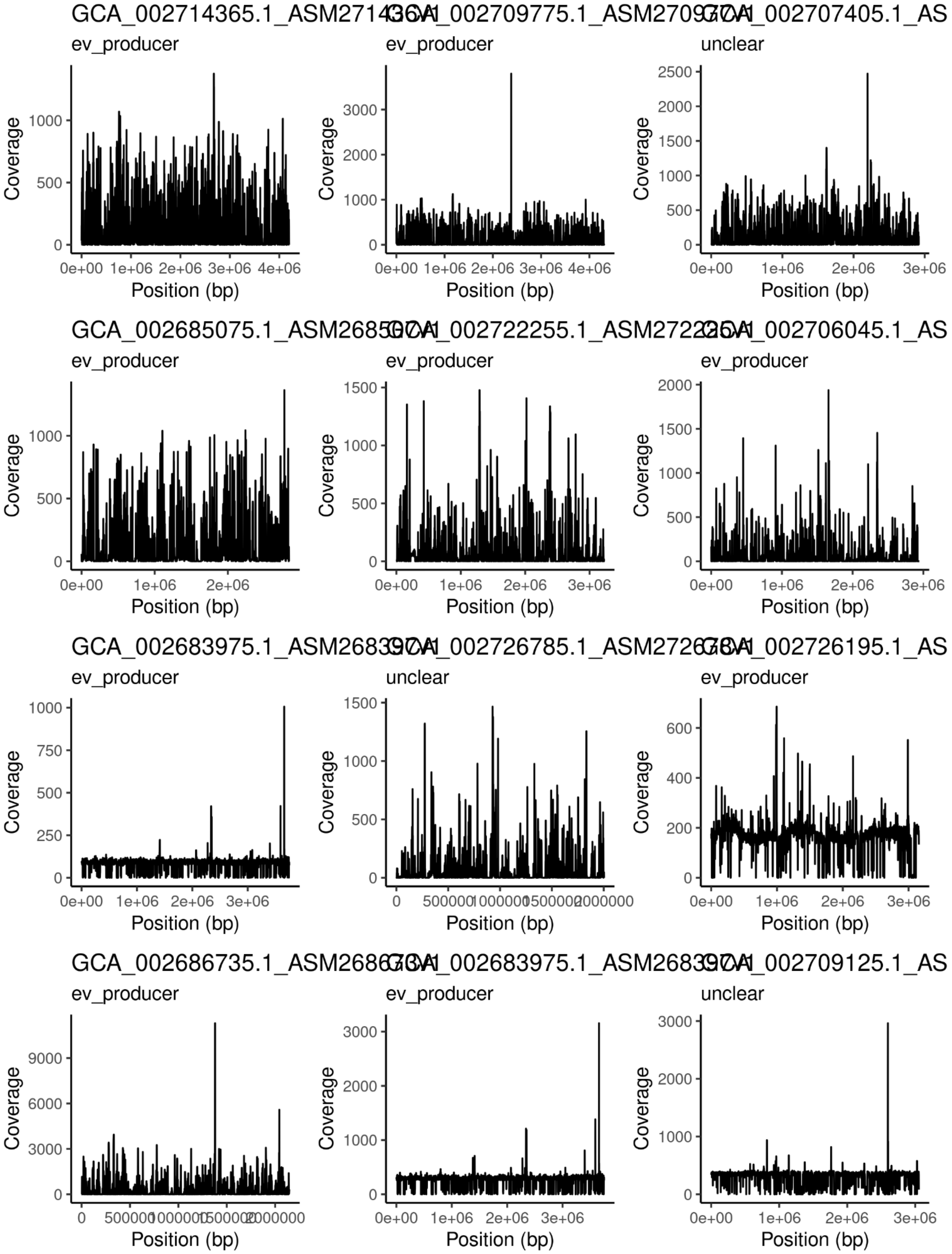

GCA_002714365.1_ASM2714
ev_producer
GCA_002709775.1_ASM2709
ev_producer
GCA_002707405.1_AS
unclear
GCA_002685075.1_ASM2685
ev_producer
GCA_002722255.1_ASM2722
ev_producer
GCA_002706045.1_AS
ev_producer
GCA_002683975.1_ASM2683
ev_producer
GCA_002726785.1_ASM2726
unclear
GCA_002726195.1_AS
ev_producer
GCA_002686735.1_ASM2686
ev_producer
GCA_002683975.1_ASM2683
ev_producer
GCA_002709125.1_AS
unclear
Coverage
Position (bp)

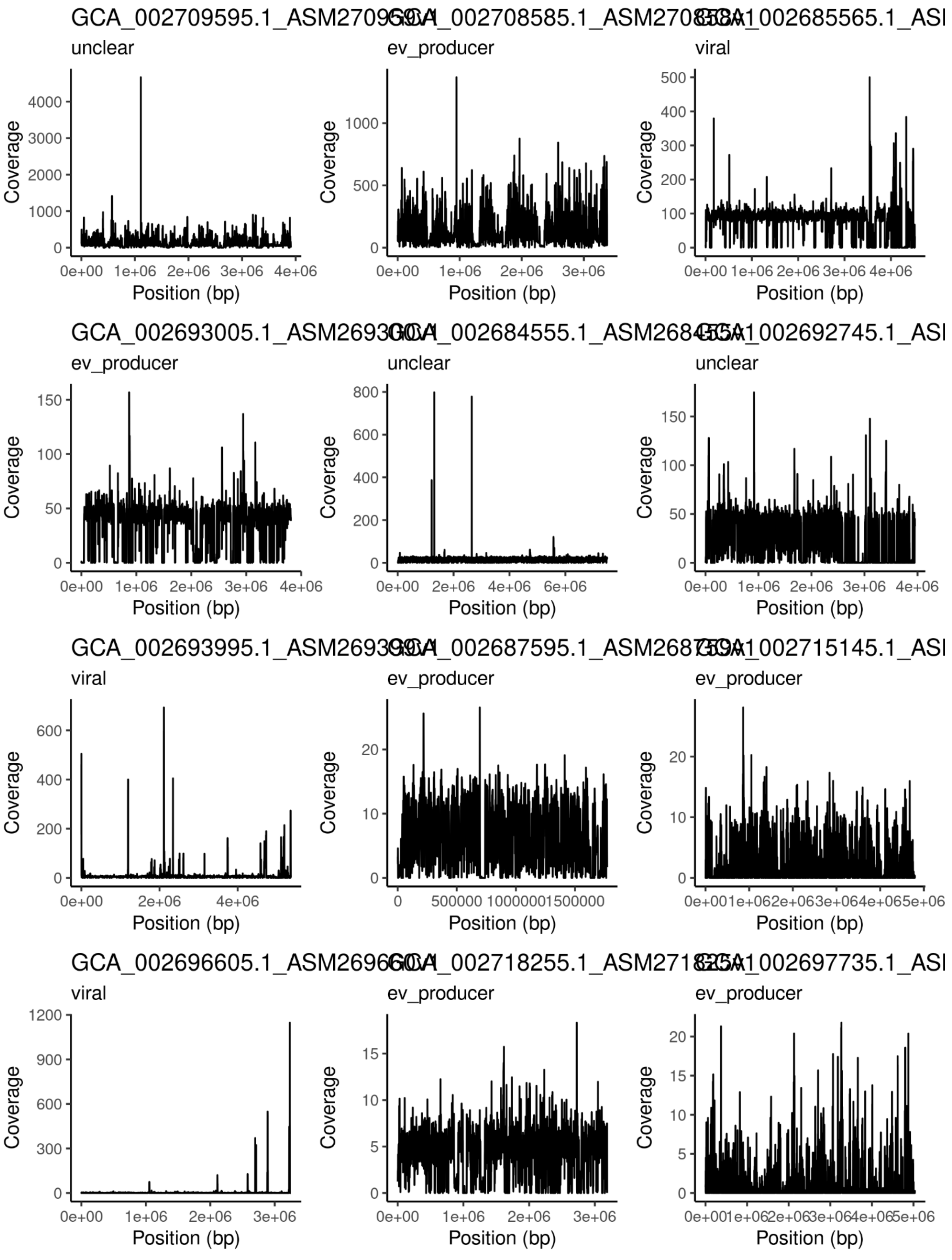
GCA_002709595.1_ASM2709
unclear
GCA_002708585.1_ASM2708
ev_producer
GCA_002685565.1_ASI
viral
GCA_002693005.1_ASM2693
ev_producer
GCA_002684555.1_ASM2684
unclear
GCA_002692745.1_ASI
unclear
GCA_002693995.1_ASM2693
viral
GCA_002687595.1_ASM2687
ev_producer
GCA_002715145.1_ASI
ev_producer
GCA_002696605.1_ASM2696
viral
GCA_002718255.1_ASM2718
ev_producer
GCA_002697735.1_ASI
ev_producer
Coverage
Position (bp)

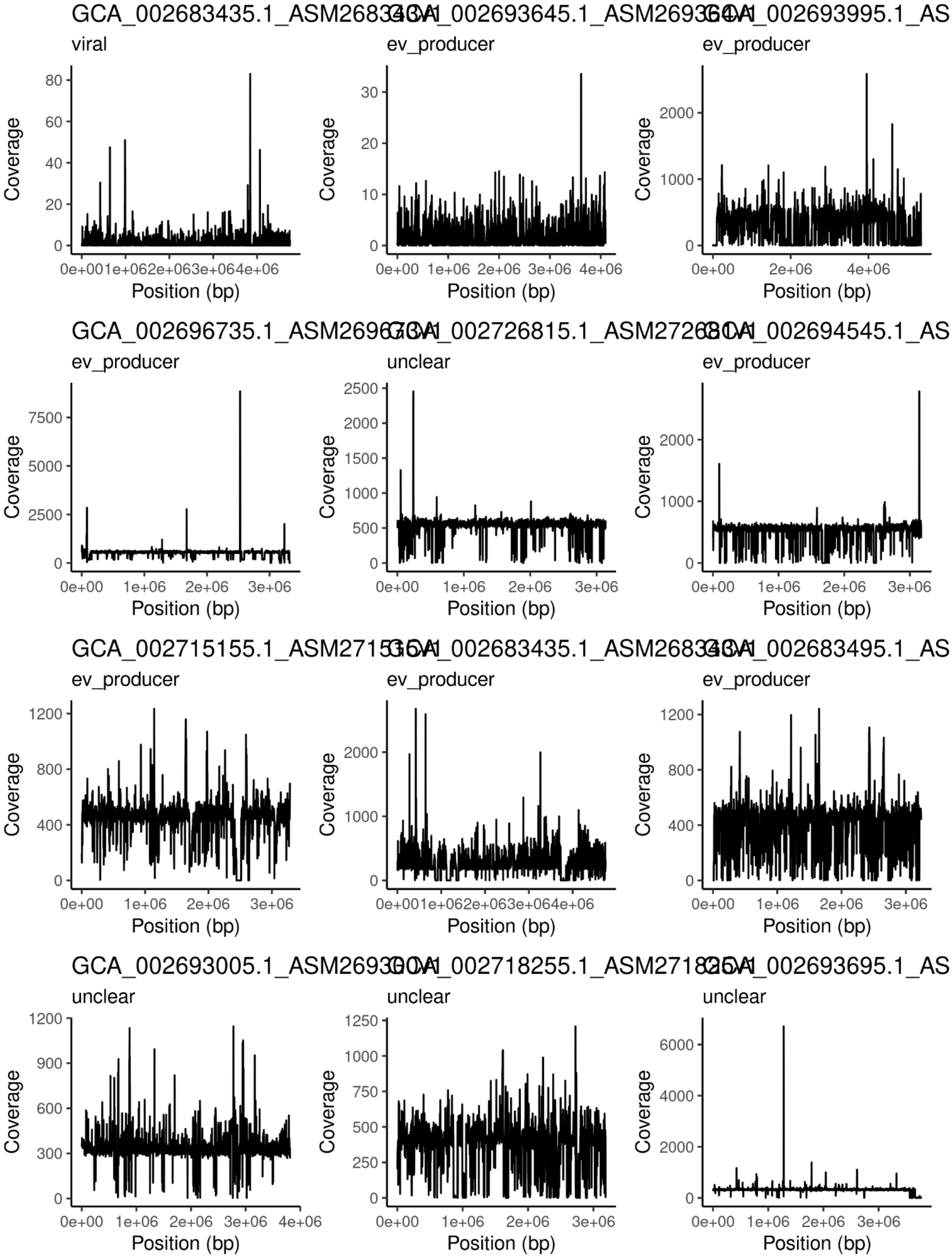

GCA_002683435.1_ASM2683
GCA_002693645.1_ASM2693
GCA_002693995.1_AS
viral
ev_producer
ev_producer
GCA_002696735.1_ASM2696
GCA_002726815.1_ASM2726
GCA_002694545.1_AS
ev_producer
unclear
ev_producer
GCA_002715155.1_ASM2715
GCA_002683435.1_ASM2683
GCA_002683495.1_AS
ev_producer
ev_producer
ev_producer
GCA_002693005.1_ASM2693
GCA_002718255.1_ASM2718
GCA_002693695.1_AS
unclear
unclear
unclear
Coverage
Position (bp)

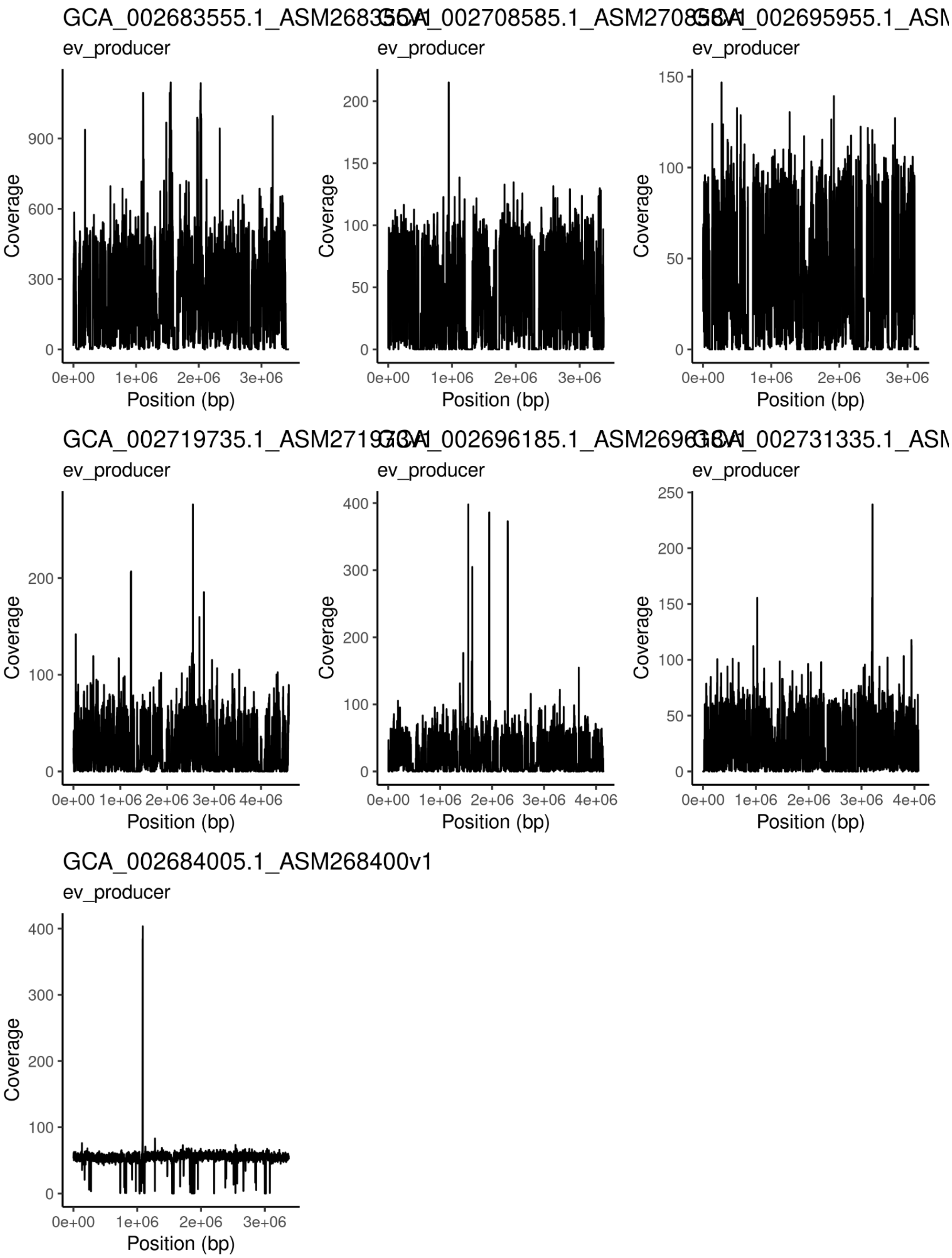

GCA_002683555.1_ASM2683
GCA_002708585.1_ASM2708
GCA_002695955.1_ASM
ev_producer
Coverage
Position (bp)
GCA_002719735.1_ASM2719
GCA_002696185.1_ASM2696
GCA_002731335.1_ASM
GCA_002684005.1_ASM268400v1
